# The Effect of Oil-Rich Food Waste Substrates, Used as an Alternative Carbon Source, on the Cultivation of Microalgae—A Pilot Study

**DOI:** 10.3390/microorganisms11071621

**Published:** 2023-06-21

**Authors:** Pavlína Sniegoňová, Martin Szotkowski, Jiří Holub, Pavlína Sikorová, Ivana Márová

**Affiliations:** Faculty of Chemistry, Brno University of Technology, 61200 Brno, Czech Republic

**Keywords:** microalgae, cyanobacteria, waste frying oil, coffee oil, metabolites, lipids

## Abstract

Microalgae are mostly phototrophic microorganisms present worldwide, showcasing great adaptability to their environment. They are known for producing essential metabolites such as carotenoids, chlorophylls, sterols, lipids, and many more. This study discusses the possibility of the mixotrophic abilities of microalgae in the presence of food waste oils. The utilization of food waste materials is becoming more popular as a research subject as its production grows every year, increasing the environmental burden. In this work, waste frying oil and coffee oil were tested for the first time as a nutrition source for microalgae cultivation. Waste frying oil is produced in large amounts all over the world and its simple purification is one of its greatest advantages as it only needs to be filtered from leftover food pieces. Coffee oil is extracted from waste spent coffee grounds as a by-product. The waste frying oil and coffee oil were added to the basic algal media as an alternative source of carbon. As a pilot study for further experimentation, the effect of oil in the medium, algal adaptability, and capability to survive were tested within these experiments. The growth and production characteristics of four algae and cyanobacteria strains were tested, of which the strain *Desmodesmus armatus* achieved exceptional results of chlorophyll (8.171 ± 0.475 mg/g) and ubiquinone (5.708 ± 0.138 mg/g) production. The strain *Chlamydomonas reindhartii* showed exceptional lipid accumulation in the range of 30–46% in most of the samples.

## 1. Introduction

Microalgae are photosynthetic eukaryotic organisms inhabiting mostly aquatic environments, both freshwater and saltwater. One of their main features is the fixation of carbon dioxide during the process of photosynthesis while producing oxygen [1,2,3]. Using carbon dioxide as a main source of energy is most common, however, some mixotrophs are capable of the occasional usage of organic carbon sources to produce energy [1,4]. Microalgae are present worldwide and they are able to adapt to more difficult environments [5,6]. They are able to survive in extreme conditions such as high temperatures, high salinity, the presence of high concentrations of metals and even combinations of these conditions, and more [5]. Microalgal biomass is rich in valuable bioactive metabolites such as proteins, carbohydrates, lipids, polysaccharides, pigments, etc. As a rich source of fiber, vitamins and minerals, microalgae have found wide application in the food industry, especially as nutritional supplements and animal feed additives [7,8,9]. The production of biologically active substances makes them great for potential use in the pharmaceutical industry [9,10,11]. As the issue with fossil fuels as energy resources grows more important, the research has focused on using algae as a biofuel source, mostly the production of biodiesel from microalgae [3,12,13,14].

With a growing population, the demand for food production increases. This trend has seen the amount of food waste increase yearly [15] (estimated at 1.3 billion tons annually) [16,17]. Food waste is either disposed into landfills or can be treated otherwise. One of the options is using food waste as feedstock in biotechnology processes [17]. Food waste is rich in organic compounds such as carbohydrates, fats, nitrogenous compounds, vitamins, minerals, etc., which can be used as a source of organic carbon for microorganisms [16]. 

The coffee industry is growing each year due to the enormous popularity of the beverage and coffee products. During the process of brewing coffee or making instant coffee, a large amount of waste in the form of spent coffee grounds (SCG) is generated [18,19,20]. Spent coffee grounds are still rich in organic components such as polysaccharides, lignin and proteins, oils, and many smaller components that can include phenolic compounds, pigments, etc. [21]. Depending on the type of coffee source, SCG can contain from 10 up to 20% of oil. One possible application of coffee oil is its conversion into biodiesel through the transesterification method [22].

The food industry also produces a large number of other oil-based waste products among which waste frying oil (WFO) is important, mainly coming from fast-food chains and large restaurants [23]. Not only is there a large amount of waste frying oil, but its disposal also poses an environmental problem as it is regularly poured into drains [24,25]. WFOs are highly available and relatively cheap, therefore they have been researched for biodiesel synthesis [26,27]. However, due to the chemical changes during the frying process, the quality of the biodiesel can be significantly influenced. This requires some pre-treatment of the feedstock and detailed control of the process [25,28]. 

The aim of this study was to test the ability of selected algal strains to grow mixotrophically in a basic Bold’s Basal Medium (BBM) with added food waste oils as an organic carbon source. This experimental work was conducted as a pilot study for subsequent research, mainly for yeast:microalgae co-cultivation experiments [29]. To the best of our knowledge, the present study is the first work focused on the cultivation of microalgae on oil-based food waste materials. Coffee oil was used in this study for a comparison with waste frying oil and also for the possibility of testing microalgae for the absorption of valuable substances from these oils. In our previous experiments with yeast, the high capacity of yeast to absorb valuable substances from wastes such as tocopherol from coffee oil was confirmed.

## 2. Materials and Methods

### 2.1. Microalgae Strains

Based on the results of our previous experiments and published results [29], four algae and cyanobacteria strains were enrolled in this study as follows: *Desmodesmus armatus* CCALA 439, *Scenedesmus acutus* CCALA 904, *Chlamydomonas reindhartii* CCALA 973, and *Anabaena torulosa* CCALA 3. All strains were purchased from the Culture Collection of Autotrophic Organisms of the Institute of Botany of the Czech Academy of Sciences, Třeboň, Czech Republic. Strains were preserved on slant agar tubes with Bold’s Basal Medium (BBM).

### 2.2. Media Preparation

All strains used were cultivated on a standard BBM medium enriched with several lipid waste materials that served as carbon sources. The used lipid waste materials were waste frying oil (WFO) and coffee oil (CO). Waste frying oil (sunflower oil) was obtained from the household kitchen. The leftover pieces of food and other particles were filtered through filtration paper under a vacuum. The coffee oil was obtained from SCG and collected from the university kitchen. The oil is a by-product of SCG hydrolysis and was extracted with hexane via Soxhlet extraction and subsequently evaporated under vacuum. Both oils were then sterilized and stored in the fridge at 4 °C. The final medium composition per liter was: 0.250 g NaNO_3_, 0.075 g MgSO_4_·7H_2_O, 0.025 g CaCl_2_·2H_2_O, 0.006 g citric acid, 0.075 g K_2_HPO_4_, 0.175 g KH_2_PO_4_, 0.025 g NaCl, 0.050 g EDTA, 0.0049 g FeSO_4_, 0.0310 g KOH, 0.0088 g ZnSO_4_·7H_2_O, 0.0014 g MnCl_2_·4H_2_O, 0.0007 g MoO_3_, 0.0016 CuSO_4_·5H_2_O, and 0.0005 g Co(NO_3_)_2_·6H_2_O. The final pH of the BBM media was adjusted to the final pH = 6.6 using HCl. The amount of waste oil in the media per 50 mL was: Control 0 g, WFO/CO 50 0.585 g, WFO/CO 75 0.878 g, WFO/CO 100 1.170 g, WFO/CO 125 1.463 g, and WFO/CO 150 1.756 g (Table 1). The amount was calculated according to our previous co-cultivation experiments and the amount of lipid substrate in yeast cultivation media (100 is a standard concentration). The fatty acid profile and total lipid analysis are shown in Table 2 [29]. The groups were divided into saturated fatty acids (SFA), monounsaturated fatty acids (MUFA), and polyunsaturated fatty acids (PUFA). 

### 2.3. Cultivation Conditions

The experiments were performed in Erlenmeyer flasks on orbital shakers for the duration of 21 days. Laboratory temperature: 23 °C; media volume: 50 mL; light intensity: 180 µmol·m^−2^·s^−1^ of photons; light/dark period: 16/8 h; carbon dioxide input: no additional input; shaking: orbital, 110 rpm.

### 2.4. Analytical Methods

#### 2.4.1. Cell Dry Weight

Media samples with grown culture (50 mL) were centrifuged at 7000 rpm for 4 min. The supernatant was collected for further analyses (waste oil, pH) and stored at −20 °C. Then, the microalgae cells were washed twice with the mixture of distilled water and hexane 4:1 (*v*/*v*) and suspended in 1 mL of distilled water. Next, the purified microalgae biomass was transferred quantitatively into Eppendorf tubes, frozen at −35 °C and freeze-dried. After determining the weight to calculate the CDW, the dried biomass was stored at −20 °C for further analyses of carotenoids, ergosterol, ubiquinone, lipids, and glucans.

#### 2.4.2. Pigment Analysis

The total carotenoid, coenzyme Q, sterol, and chlorophyll content were determined using HPLC-DAD chromatography. Samples of freeze-dried algae biomass (approx. 10–15 mg) were rehydrated with 1 mL of MiliQ water for 60 min. The water excess was removed by centrifugation at 10,000 rpm for 3 min, discarded, and 1 mL of methanol and about 0.5 mL of glass beads (0.2–0.5 mm diameter) were added to the sample. The sample was homogenized in a laboratory homogenizer for 3 × 30 s and then quantitatively transferred to a 15 mL centrifuge tube and washed with 2 mL of chloroform. The mixture was further vortexed for 10 min. Then, 1 mL of water was added, the mixture was vortexed for 15 s, and the tube was allowed to stabilize for two phases. 

The lower chloroform phase was quantitatively transferred to a clean tube and dried under an inert nitrogen atmosphere. The dried sample was dissolved in 2 mL of 2:1 EtAc:ACN and filtered through a 0.45 μm PTFE filter into the vial. Samples were measured on a Dionex Ultimate series HPLC with a Vanquish DAD detector (Thermo Fischer Scientific, Waltham, MA, USA) on a Kinetex C18-EVO column 150 mm × 4.6 mm × 5 µm (Phenomenex, Torrance, CA, USA) using gradient separation with mobile phase A (ACN:MeOH:Tris HCl pH = 8; 84:2:14) and mobile phase B (MeOH:EtAc = 60:40) at the flow rate of 1.2 mL/min and 25 °C. The gradient program uses 100% of mobile phase A from the start. The gradient is changed at the thirteenth minute and 100% of mobile phase B is used until minute 20. Then, until the end of the analysis, the program again uses 100% of mobile phase A. Carotenoid pigments were detected at 445 nm and chlorophylls at 445 nm and 455 nm, respectively. Chromatographic data were evaluated using Chromeleon 7.2. software. The total carotenoid, sterol, coenzyme Q, and chlorophyll production were identified and evaluated using commercial standards (Sigma Aldrich, St. Louis, MO, USA) and external calibration as previously described [29,30].

#### 2.4.3. Lipids and Fatty Acid Analysis

The total lipids and individual fatty acids were determined by optimized GC/FID analysis. The lipids and fatty acid analyses were performed to determine the percentage of produced lipids as well as the profile (percentage of each group) of fatty acids. Approximately 5–10 mg of the freeze-dried microalgae biomass was weighed into a 2.0 mL crimp neck vial together with 1.8 mL 15% (*v*/*v*) H_2_SO_4_ in methanol, capped with an aluminum cap and heated at 85 °C for 2 h. After finishing the transesterification process, the sample was transferred quantitatively into a 5 mL vial and neutralized with 0.5 mL of 0.005 M NaOH. The FAMEs mixture was converted to the non-polar phase by adding 1 mL of n-hexane and shaking vigorously. The total lipid content and fatty acid profile were determined by gas chromatography/flame ionization detection (GC/FID) analysis. GC analysis of FAMEs was carried out on a TRACETM 1300 gas chromatograph (Thermo Fischer Scientific, USA) equipped with a flame ionization detector, an Al 1310 autosampler, and a Lion GC-FAME column (30 m, 0.25 mm, 0.20 μm) (Chromservis, Prague, Czech Republic). The temperature gradient during the analysis was set to 80 °C from the injection (tR = 1 min) and was increased at a rate of 15 °C·min^−1^ up to 140 °C (tR = 5 min), then increased by a rate of 3 °C·min^−1^ up to 190 °C (tR = 21.7 min). From that point on, the growth rate was increased at a rate of 25 °C·min^−1^ until it reached a temperature of 260 °C, where this temperature was maintained for 1 min, at which point the program stopped at tR = 25.5 min. FAMEs were identified using the commercial standard Supelco 37 Component FAME Mix (Sigma Aldrich, SRN). The internal standard method was used for quantification via the addition of heptadecanoic acid (Sigma Aldrich, SRN) into the transesterification mixture in the concentration of 0.5 mg/mL. Chromatography data were evaluated using Chromeleon software 7.2 [31]. Based on the GC-FID analysis, representation of the fatty acids was analyzed via the Chromeleon program. The percentage of individual types of fatty acids was calculated from their sum relative to the total number of fatty acids.

### 2.5. Statistical Analysis

The growth experiments in Erlenmeyer flasks were carried out in triplicate. The presented results are the mean of the replicates and the standard deviations are shown as error bars in the figures. Data handling and statistics were performed using the Excel software package (Microsoft Excel 2013, Microsoft Corp., Redmond, WA, USA). The experimental data from the pilot study were also subjected to analysis via the Statistica program (Stanford, CA, USA). The analysis focused on the correlation between the amount of oil in the media and the production of biomass and lipids. To determine the normal distribution of data, the Shapiro–Wilk test was performed. Based on the results, either ANOVA or the Kruskal–Wallis method was implemented to determine any statistical difference in the analyzed data (*p*-value set to 0.05).

## 3. Results

In this study, we present the first published results that examined the possible growth of microalgae on oil waste substrates. Based on our previous experiments [29], cultivations were conducted to determine whether the microalgae cultures were able to grow in simple BBM with the addition of different amounts of waste frying oil and coffee oil. The main goal was to determine whether the studied strains were able to utilize waste lipid substrates, and thus to incorporate some molecules from used oils to the biomass. We aimed to verify whether mixotrophy growth could be used in co-cultivation experiments of microalgae with heterotrophic microorganisms such as red yeasts to produce a combined yeast:microalgae enriched biomass that could be used as a food and feed additive [7]. In the experiments, the growth rate of microalgae in the control media was monitored and compared to the media with added oils. In the case where the microalgae culture was not able to utilize the added oils, it was necessary to monitor whether the presence of the oil had a negative effect on the viability of the microalgae, or whether it inhibited photosynthesis by obstructing the light availability and thus the absorption of light by cells. Different concentrations of the tested oils were then used to identify the limits of tolerance of the microalgae to the oil, as this parameter is very important for subsequent co-cultivation experiments.

### 3.1. Desmodesmus Armatus CCALA 439

**Figure 1:**
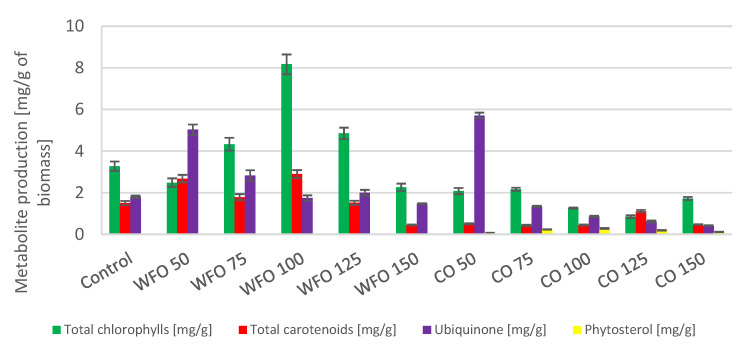
Lipid metabolite HPLC analysis of *Desmodesmus armatus* cultivated on BBM with different oil concentrations.

Cultivation of *Desmodesmus armatus* with the addition of waste frying oil showed a higher production of total chlorophylls compared to the control except for WFO 50 and WFO 150. The highest production of total chlorophylls was obtained with WFO 100 (8.171 ± 0.475 mg/g). The production of total carotenoids did not seem to depend on the concentration of oil present in the medium. Apart from the highest concentration, WFO 150, the production of carotenoids was higher compared to the control. The best production of carotenoid pigments (Figure 1) was found in sample WFO 100 (2.901 ± 0.188 mg/g). In the case of ubiquinone, the production in sample WFO 50 was greatly increased (5.031 ± 0.246 mg/g) compared to the control, and an observable trend of lowering its concentration with increasing concentrations of oil in the medium was present. Only in sample WFO 150 was a lower production observed than in the control. Overall, sample WFO 100 showed a higher production of targeted metabolites compared to the control. In sample WFO 150, the production decreased considerably compared to the control.

Regarding the samples cultivated with the addition of coffee oil, the production of metabolites was decreased compared to the control and samples cultivated with WFO. Except for ubiquinone, all metabolite production was lower than the control. The greatest production could be seen in sample CO 50 (5.708 ± 0.138 mg/g), which was also higher than in sample WFO 50. With higher concentrations of CO, the production of ubiquinone decreased more than in the WFO samples. Within the samples cultivated in the presence of coffee oil, the production of total carotenoids was significantly higher in the sample CO 125. The best production of total chlorophylls was in sample CO 75 (2.167 ± 0.075 mg/g). The samples cultivated on coffee oil showed a slight production of sterols, with the best production in CO 100 (0.284 ± 0.022 mg/g).

The cultivation in the presence of oil showed a positive effect on the production of biomass with the exception of sample CO 150, where the yield was lower than the control. The biomass yield in samples cultivated on WFO was slightly higher overall than in the samples cultivated on CO, with sample WFO 150 displaying the greatest production of biomass (1.134 ± 0.099 g/L) (Table 3). The Shapiro–Wilk test for normality determination was applied to analyze the data for biomass production. This parameter did not show a normal distribution, therefore the Kruskal–Wallis method was applied (*p* = 0.8804).

**Figure 2:**
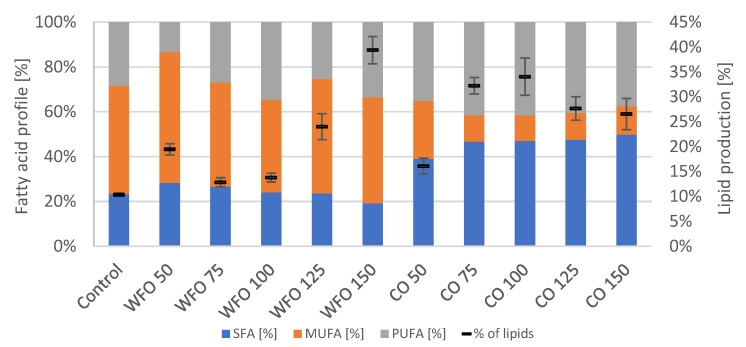
Fatty acid profile (left y axis) and lipid production (right y axis) of *Desmodesmus armatus* cultivated on BBM with different oil concentrations.

The GC analysis of lipid production showed (Figure 2) that cultivation with the addition of oils did increase the production of total lipids compared to the control. For WFO, the lipid production increased with the higher concentration of oil with the exception of WFO 50. The highest lipid production was recorded in sample WFO 150 (39.38 ± 2.74%). The profile of fatty acids was not significantly different among the samples cultivated on WFO and the control, which had the highest representation of monounsaturated fatty acids (MUFA) (above 40%); the highest content was in sample WFO 50 (58.39%). The content of SFA ranged from about 19% to 28%. The least amount of polyunsaturated fatty acids (PUFA) was in sample WFO 50 (13.36%).

The samples cultivated with coffee oil also showed higher lipid production than the control with the lowest production in sample CO 50, then increased with higher concentrations ranging from 16 to 34%. The fatty acid composition in samples cultivated with coffee oil did not differ significantly among each other with the slight exception of sample CO 50. The samples cultivated on coffee oil showed the highest representation of saturated fatty acids (SFA) (above 40%) and the lowest for MUFA (approx. 11–13%).

The Shapiro–Wilk test for normality determination was also applied to analyze the data for lipid production. This parameter also did not show a normal distribution, therefore, the Kruskal–Wallis method was applied for lipid production (*p* = 0.2932).

### 3.2. Scenedesmus Acutus CCALA 904

**Figure 3:**
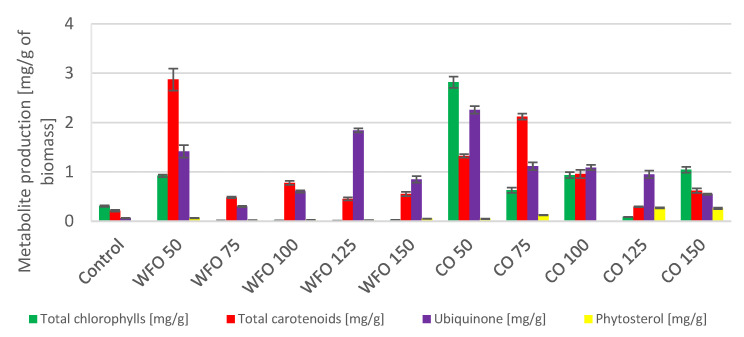
Lipid metabolite HPLC analysis of *Scenedesmus acutus* cultivated on BBM with different oil concentrations.

Microalgae cultivated on frying oil displayed a decrease in the production of total chlorophylls, except for WFO 50. This sample showed the greatest production of chlorophylls (0.920 ± 0.025 mg/g) as well as total carotenoids (2.872 ± 0.224 mg/g). The total carotenoid production decreased with higher concentrations of oil but these productions were higher than that of the control. A considerable increase (Figure 3) was observed in the production of ubiquinone, where the best production was in sample WFO 125 (1.842 ± 0.040 mg/g). The production of certain metabolites was higher in samples cultivated with added oil compared to the control, however, the productions of chlorophylls and carotenoids were drastically decreased, possibly by the degradation of the mentioned metabolites.

In the case of coffee oil, the addition did not have such a negative effect on the production of total chlorophylls as waste frying oil. Therefore, the production of metabolites was also increased compared to the control. The production of chlorophylls was the highest in sample CO 50 (2.818 ± 0.112 mg/g), and the production in other samples was also rather high compared to the control, with the exception of sample CO 125. The production of total carotenoids was predominantly higher for the three lowest concentrations of coffee oil. The highest carotenoid production was seen in sample CO 75 (2.121 ± 0.062 mg/g). For ubiquinone, the best production was found in sample CO 50 (2.257 ± 0.076 mg/g) and decreased as concentrations of oil in the medium was increased. With the addition of coffee oil in the medium, the production of sterols was slightly increased, and the highest values were measured in sample CO 125 (0.270 ± 0.010 mg/g). The coffee oil did not have a negative effect on the production of chlorophylls and other carotenoids (pigments), while the frying oil showed a negative effect with the exception of culture sample WFO 50.

**Figure 4:**
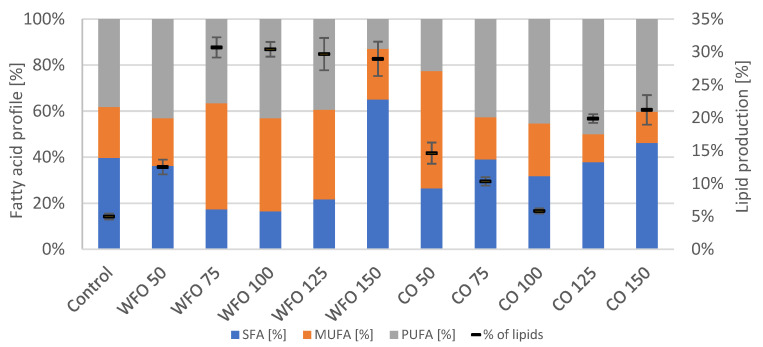
Fatty acid profile (left y axis) and lipid production (right y axis) of *Scenedesmus acutus* cultivated on BBM with different oil concentrations.

In the case of algae *Scenedesmus acutus*, the addition of oil had a positive effect on the production of biomass for both WFO and CO. The greatest production was achieved by samples WFO 100 (1.190 ± 0.085 g/L) and CO 150 (1.214 ± 0.149 g/L) (Table 4). Data from strain *Scenedesmus acutus* were subjected to the Shapiro–Wilk test, which did not show a normal data distribution. The Kruskal–Wallis test was then applied for biomass production (*p* = 0.2606). Analysis of the total amount of lipids in the biomass showed an increased production of lipids in all samples cultivated with oil compared to the control. The samples cultivated on waste frying oil showed production ranging from approx. 29% to 31%, with the exception of sample WFO 50 (12.5 ± 1.11%). The samples cultivated on coffee oil showed a decrease in the production of lipids with an increasing concentration of oil until sample CO 100, then increased rapidly in the case of the two highest concentrations of oil. The highest lipid content (Figure 4) was found in sample CO 150 (21.19 ± 2.25%). In comparison with the waste frying oil, the samples cultivated on coffee oil did not reach that high amount of lipids. The data for lipid production were subjected to the Shapiro–Wilk test, which did not show a normal distribution of data. The Kruskal–Wallis test was then applied (*p* = 0.4297).

The fatty acid composition did not seem to follow a united trend for all samples (Figure 4). However, with the exception of CO 50, the samples cultivated on coffee oil produced the least amount of MUFA (approx. 12–23%), while the highest production varied between SFA and PUFA.

### 3.3. Chlamydomonas Reindhartii CCALA 973

**Figure 5:**
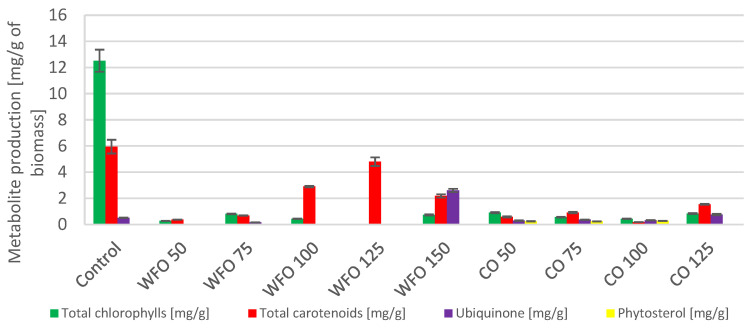
Lipid metabolite HPLC analysis of *Chlamydomonas reindhartii* cultivated on BBM with different oil concentrations.

Microalgae *Chlamydomonas reindhartii* displayed a severe formation of emulsion (Figure S1). A significant decrease in the production of total chlorophylls as well as other metabolites was observed in this strain (Figure 5). Among the samples cultivated on waste frying oil, sample WFO 75 showed the greatest production of total chlorophylls (0.812 ± 0.034 mg/g). The production of carotenoids was higher in the samples also containing a higher amount of WFO in the medium, with the best production in sample WFO 125 (4.802 ± 0.327 mg/g). The only significant production of ubiquinone was seen in sample WFO 150 (2.607 ± 0.132 mg/g), which was also higher compared to the control. Samples cultivated on coffee oil showed similar results, especially a significant decrease in the production of metabolites (pigments). Among the samples, the highest production of total chlorophylls was in sample CO 50 (0.907 ± 0.053 mg/g). Sample CO 125 achieved the highest production of ubiquinone (0.764 ± 0.060 mg/g). Additionally, a slight production of sterols could be seen in the samples cultivated on coffee oil. The production of chlorophylls and carotenoids was severely decreased, possibly due to light obstruction during cultivation.

Microalgae samples cultivated on both WFO and CO displayed an increase in the biomass production compared to the control. The biomass production differed in individual samples and was not dependent on parameters of the cultivation. Sample WFO 75 displayed the greatest biomass yield (1.376 ± 0.096 g/L) (Table 5) within the samples cultivated on WFO. Overall, the highest biomass production was found in sample CO 75 (1.496 ± 0.182 g/L). The biomass production data were subjected to the Shapiro–Wilk test, which determined a normal data distribution. Therefore, the ANOVA method was performed (*p* = 0.000791).

**Figure 6:**
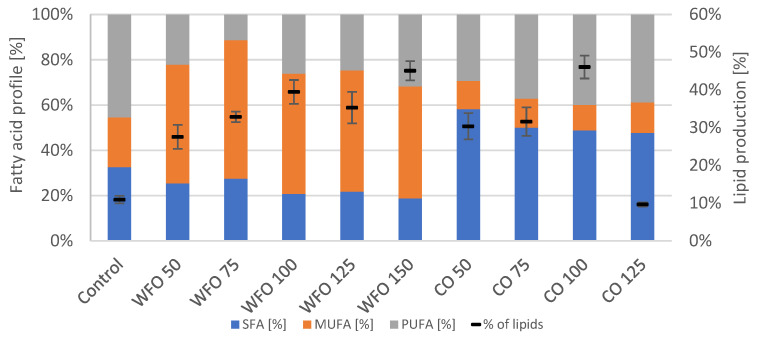
Fatty acid profile (left y axis) and lipid production (right y axis) of *Chlamydomonas reindhartii* cultivated on BBM with different oil concentrations.

Unlike the HPLC analysis, the GC analysis (Figure 6) showed an increased production of lipids in all samples cultivated in the presence of oil with the exception of sample CO 125 (9.67 ± 0.54%), which was slightly lower compared to the control (10.94 ± 0.99%). The highest production of lipids could be seen in sample CO 100 (46.07 ± 3.03%) and WFO 150 (45.09 ± 2.54%). The production of lipids in samples cultivated on waste frying oil showed an increase at higher concentrations of oil in the medium. Regarding the representation of fatty acids in the lipidic part, the content of each group seemed to be similar among the samples cultivated on the same type of oil (Figure 6). The samples cultivated on waste frying oil showed a very high production of MUFA (above 49%). The Shapiro–Wilk test for lipid production did not show a normal distribution of data. The Kruskal–Wallis test was performed (*p* = 0.0212). 

The highest level of MUFA was observed in sample WFO 75 (61.14%). The least represented group was either the SFA or PUFA. The lowest production of PUFA was also seen in sample WFO 75 (11.34%). Samples cultivated on coffee oil had the highest SFA (approx. 47–59%) and lowest MUFA production (up to 14%). This fatty acid profile was more similar to the control than the profile of the samples cultivated on waste frying oil.

### 3.4. Anabaena Torulosa CCALA 3

**Figure 7:**
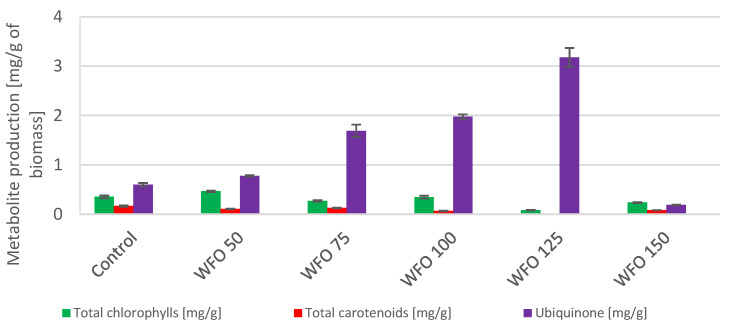
Lipid metabolite HPLC analysis of *Anabaena torulosa* cultivated on BBM with different oil concentrations.

The presence of coffee oil in the media showed a negative effect on the viability of cultures of the strain *Anabaena torulosa* and caused their death. Cyanobacteria samples cultivated on WFO displayed a decrease in the production of chlorophylls (Figure 7) with the exception of sample WFO 50 (0.464 ± 0.015 mg/g), whose yield was higher compared to the control. Regarding the total carotenoids, the highest production among the samples cultivated in the presence of oil was found in sample WFO 75 (0.126 ± 0.006 mg/g), which was, however, lower compared to the control. The production of ubiquinone displayed a higher yield in samples cultivated on oil compared to the control and increased with higher concentrations of oil in the medium. Sample WFO 125 displayed the greatest ubiquinone production (3.177 ± 0.191 mg/g) with the exception of sample WFO 150 (0.187 ± 0.008 mg/g), where the production was decreased significantly. 

**Figure 8:**
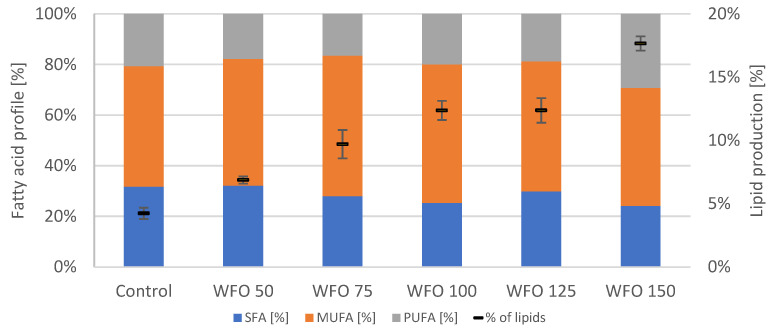
Fatty acid profile (left y axis) and lipid production (right y axis) of *Anabaena torulosa* cultivated on BBM with different oil concentrations.

Concerning this strain, the addition of oil did not have any significant effect on the production of biomass. Sample WFO 50 displayed the greatest biomass yield (0.520 ± 0.026 g/L) (Table 6). The production of lipids (Figure 3) grew with increasing concentrations of oil in the medium, with the best production in sample WFO 150 at 17.66 ± 0.57%. The profile of fatty acids did not significantly differ from the control (Figure 8). The highest portion were MUFA ranged from approx. 47% to 56%, while the PUFA were represented the least, ranging from approx. 16% to 30%. 

Triplicates were performed on this strain, however, throughout the tests, many of the cultures did not survive. Therefore, from the obtained data, we were not able to perform the ANOVA analysis. These tests will be repeated in the near future.

## 4. Discussion

In the present work, some microalgae were cultivated in the presence of different concentrations of either waste frying oil or coffee oil. The main focus of this experiment was to determine the viability and growth of the cultures in the presence of an oil-based nutrition source in the media, as this experiment was conducted as a pilot study for further experiments. Furthermore, the effect of the oil on the production of some specific algae metabolites was analyzed. The presence of oil in the medium caused the creation of emulsion with different severity. With the more severe creation of an emulsion in the medium, the obstruction of light absorption was also more severe. As a consequence, a negative effect on the production of pigments was observed, and simultaneously, an increase in the production of lipids was observed. Additionally, the darker color of coffee oil could have negative effect on the light transmission into the medium.

Cultivation of the microalgae *Desmodesmus armatus* CCALA 439 resulted in a decreased production of total chlorophylls and carotenoids in media with added coffee oil. The production of ubiquinone was the highest for the samples cultivated in the presence of the lowest concentrations of both waste frying oil and coffee oil (WFO 50, CO 50), with the greatest production in sample CO 50 (5.708 ± 0.138 mg/g). A significant increase in the production of total chlorophylls was observed in sample WFO 100 (8.717 ± 0.475 mg/g). Regarding the production of total carotenoids concerned, a noticeable increase was observed in samples WFO 50 and WFO 100, with the greatest production in sample WFO 100 (2.901 ± 0.788 mg/g). For the samples cultivated in the presence of coffee oil in the media, the production of the metabolites was mostly decreased compared to the control. As above-mentioned, with the decreased production of pigments in the samples cultivated on coffee oil, the production of lipids did increase. A noticeable increase in the production of lipids in the samples cultivated on WFO was also observed to have the greatest production of lipids, as shown in sample WFO 150 (39.38 ± 2.74%). The explanation is probably complex and except for the influence on photosynthesis includes at least (i) the oxidation of nutrients to acetyl-CoA as a precursor of both isoprenoids and lipids and (ii) the incorporation of lipids from media into the cell biomass. 

The microalgae *Scenedesmus acutus* CCALA 904 showed a production of lower amounts of total chlorophylls and carotenoids in samples cultivated on waste frying oil compared to the samples cultivated on coffee oil. The greatest production of total carotenoids could be seen in sample WFO 50 (2.872 ± 0.224 mg/g). For sample CO 50, the highest production of total chlorophylls (2.818 ± 0.112 mg/g) and ubiquinone (2.257 ± 0.076 mg/g) could be observed. Within the samples cultivated on coffee oil, the production of phytosterols was noticed, with the highest production in sample CO 125 (0.270 ± 0.010 mg/g). The production of metabolites was the opposite to the previous algal strain, which is probably a result of a different formation of an emulsion within the samples. The production of lipids followed this trend, where the production of lipids was higher in samples with a lower production of pigments, in this case, the samples cultivated on waste frying oil. This result is similar to the previous example and is also similar to the metabolic changes of some carotenogenic yeasts grown on fat media [30]. In *S. acutus*, the greatest production was observed in sample WFO 75, with a lipid yield above 30%.

In samples of microalgae *Chlamydomonas reindhartii* CCALA 973, the creation of an emulsion was very strong, regardless of the used oil, resulting in a significant decrease in the production of pigments. The greatest production of total carotenoids was observed in sample WFO 125 (4.802 ± 0.327 mg/g). For the metabolite ubiquinone, the greatest production could be seen in the sample with the highest concentration of waste frying oil, WFO 150 (2.607 ± 0.132 mg/g). Among the samples cultivated with oil in the media, the greatest production was observed in sample CO 50 (0.907 ± 0.053 mg/g), which was, however, several times lower than the production of total chlorophylls in the control sample. Samples seemed to be showing an increase in the production of lipids with increasing concentrations of oil in the media. The greatest productions of lipids were observed in samples WFO 150 (45.09 ± 2.54%) and CO 100 (46.07 ± 3.03%).

In the case of cyanobacteria *Anabaena torulosa*, the samples cultivated with coffee oil were not viable, regardless of the concentration of coffee oil used in the media. A significant increase in the production of ubiquinone was observed with increasing concentrations of waste frying oil, with the greatest production in sample WFO 125 (3.177 ± 0.191 mg/g). The production of ubiquinone could be considered as an adaptation mechanism supporting cell energy metabolism. The greatest production of total chlorophylls was observed in sample WFO 50 (0.464 ± 0.015 mg/g), which was also higher than the production of the control. The production of chlorophylls in other samples was decreased. The production of total carotenoids was also decreased compared to the control, with the greatest production in sample WFO 75 (0.126 ± 0.006 mg/g). The production of lipids increased with increasing concentrations of oil in the media.

Nowadays, lipid production by microorganisms is a rather attractive area of research and is mostly focused on microbial lipid production from organic waste and its further use (e.g., for biodiesel). Microalgae, as oleaginous microorganisms, have also been cultivated on carbon waste to produce a higher lipid content [32]. In [33], volatile fatty acids (VFAs) from low-cost organic waste were used as a majority carbon source for the cultivation of *Chlorella vulgaris*, however, the lipid production was not as high as desired. VFAs were also used as a carbon source for marine oleaginous microorganisms [34], where two freshwater algae, *A. protothecoides* and *C. sorokiniana*, were tested for their lipid production, and 28.97 and 33.79% of their cellular compartment lipids could be used as biodiesel feedstock, respectively. In another study [35], the selected carbon source was washing water rich in glycerol, where an increase of 25% in microalgae production was obtained from the cultivation of *C. vulgaris*. Other problems were also addressed such as the low oil content in microalgae cells and the higher costs of microalgae cultivation. The presentation of in situ technologies eliminates the need to extract the oil from biomass, therefore reducing the biodiesel production costs [36]. Although further research is required, other microorganisms have also been studied for their possibility to degrade and utilize waste. One of the researched fields is petroleum degradation by microorganisms (bacteria, fungus, …). Their ability to adapt to the environment and use the waste substrate as a carbon source is a very exciting opportunity for future research [37,38]. 

The microorganisms studied in this work did not show a united trend in the production of various metabolites. The production did not seem to be dependent on the used waste oil type or on the concentration of the oil in the media. The main influence on the production of metabolites and the metabolic activity as a whole seemed to be the formation of the emulsion and its severity. These effects should be further studied because there is a lack of literary data with which the results of this study can be compared.

Experiments introduced in the present study confirmed the ability of microalgae cultures to withstand the presence of either waste frying oil or coffee oil in the medium. The original metabolic strategy originated in our previous co-cultivation experiments of red yeasts and microalgae. A number of surprising, interesting, and new data regarding the production of intracellular metabolites were gained from the results. To the best of our knowledge, this study is the first to have focused on the use of oil-based waste substrates in the cultivation of microalgae. These results will provide the necessary information for further experiments and possibly new approaches in the cultivation of microalgae using waste food lipid substrates as a carbon source.

## 5. Conclusions

The results of these experiments provided us with a lot of information regarding the cultivation of microalgae in the presence of food waste oil. Emulsion creation in some samples resulted in a reduced production of pigments and other metabolites. In some pilot experiments, the results also showed a positive effect of the oil in the media on the production of several metabolites, predominantly ubiquinone and lipids. As this was a pilot experiment, there is a need for further extension and optimization in subsequent experiments. To conclude, the present pilot experiment produced a number of valuable results that represent the basis for further experiments. Furthermore, to the best of our knowledge, the pilot experiments confirm that microalgae are capable of surviving in the presence of oil in the media.

## Figures and Tables

**Table 1:** Media composition of each sample.

Sample	Composition
Control	Control BBM with no added oil
WFO/CO 50	BBM with 50% of waste frying oil/coffee oil
WFO/CO 75	BBM with 75% of waste frying oil/coffee oil
WFO/CO 100	BBM with 100% of waste frying oil/coffee oil
WFO/CO 125	BBM with 125% of waste frying oil/coffee oil
WFO/CO 150	BBM with 150% of waste frying oil/coffee oil

**Table 2:** Used waste frying oil and coffee oil analysis.

FA	WFO [%]	CO [%]
SFA	14.61	44.29
MFA	45.45	12.23
PUFA	39.94	43.49
Total lipids	94.62	93.13
Major fatty acids		
Palmitic acid	2.8 ± 0.7%	36.4 ± 1.5%
Linoleic acid	23.1 ± 1.4%	39.1 ± 0.8%
Oleic acid	43.2 ± 0.8%	10.2 ± 1.1%
Alpha-linolenic acid	10.2 ± 0.8%	1.3 ± 0.9%

**Table 3:** Biomass production by *Desmodesmus armatus*.

Biomass [g/L]					
Control	WFO 50	WFO 75	WFO 100	WFO 125	WFO 150
0.523 ± 0.016	0.714 ± 0.048	0.852 ± 0.067	0.678 ± 0.034	0.742 ± 0.035	1.134 ± 0.099
	CO 50	CO 75	CO 100	CO 125	CO 150
	0.544 ± 0.019	0.590 ± 0.020	0.718 ± 0.021	0.638 ± 0.035	0.410 ± 0.018

**Table 4:** Biomass production of *Scenedesmus acutus*.

Biomass [g/L]					
Control	WFO 50	WFO 75	WFO 100	WFO 125	WFO 150
0.080 ± 0.004	0.264 ± 0.005	1.026 ± 0.090	1.190 ± 0.085	1.060 ± 0.117	0.660 ± 0.030
	CO 50	CO 75	CO 100	CO 125	CO 150
	0.544 ± 0.021	0.390 ± 0.013	0.376 ± 0.006	0.534 ± 0.023	1.214 ± 0.149

**Table 5:** Biomass production of *Chlamydomonas reindhartii*.

Biomass [g/L]					
Control	WFO 50	WFO 75	WFO 100	WFO 125	WFO 150
0.258 ± 0.007	1.050 ± 0.091	0.1376 ± 0.096	1.248 ± 0.086	0.782 ± 0.040	1.368 ± 0.142
	CO 50	CO 75	CO 100	CO 125	
	1.432 ± 0.084	1.496 ± 0.182	1.470 ± 0.218	0.252 ± 0.006	

**Table 6:** Biomass production by *Anabaena torulosa*.

Biomass [g/L]					
Control	WFO 50	WFO 75	WFO 100	WFO 125	WFO 150
0.489 ± 0.011	0.520 ± 0.0026	0.438 ± 0.009	0.506 ± 0.025	0.500 ± 0.012	0.480 ± 0.022

## Supplementary Materials

The following supporting information can be downloaded at: https://www.mdpi.com/article/10.3390/microorganisms11071621/s1, Figure S1: Cultivation of microalgae *Chlamydomonas reindhartii* on waste frying oil.
