# Supplementary material for: The Effect of Oil-Rich Food Waste Substrates, Used as an Alternative Carbon Source, on the Cultivation of Microalgae—A Pilot Study
Source: Microorganisms. 2023 Jun 21;11(7):1621. doi: 10.3390/microorganisms11071621 (PMC10383527; doi:10.3390/microorganisms11071621)
Supplement: Supplementary file 1 [file microorganisms-11-01621-s001.zip › microorganisms-2411662-supplementary.pdf]

microorganisms

Supplementary Materials

The Effect of Oil-Rich Food Waste Substrates, Used as an Alternative Carbon Source, on the Cultivation of Microalgae—A Pilot Study

Pavlína Sniegoňová, Martin Szotkowski, Jiří Holub, Pavlína Sikorová and Ivana Márová *

Faculty of Chemistry, Brno University of Technology, 61200 Brno, Czech Republic
* Correspondence: marova@fch.vut.cz

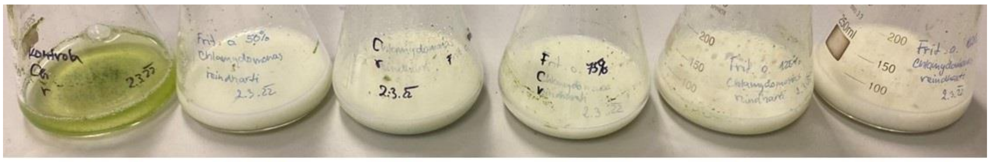

Control WFO 50 WFO 75 WFO 100 WFO125 WFO 150

Figure S1. Cultivation of microalgae *Chlamydomonas reindhartii* on waste frying oil.
